# Anti-dsDNA Antibody Isotypes in Systemic Lupus Erythematosus: IgA in Addition to IgG Anti-dsDNA Help to Identify Glomerulonephritis and Active Disease

**DOI:** 10.1371/journal.pone.0071458

**Published:** 2013-08-12

**Authors:** Danilo Villalta, Nicola Bizzaro, Nicola Bassi, Margherita Zen, Mariele Gatto, Anna Ghirardello, Luca Iaccarino, Leonardo Punzi, Andrea Doria

**Affiliations:** 1 Allergy and Clinical Immunology, Azienda Ospedaliera “S. Maria degli Angeli”, Pordenone, Italy; 2 Laboratory of Clinical Pathology, San Antonio Hospital, Tolmezzo, Italy; 3 Division of Rheumatology, Department of Medicine, University of Padova, Padova, Italy; Pavillon Kirmisson, France

## Abstract

**Objectives:**

To evaluate the role of serum IgG, IgM and IgA anti-dsDNA antibody isotypes in the diagnosis of systemic lupus erythematosus (SLE), and their association with clinical features and disease activity, in a large cohort of SLE patients.

**Methods:**

Sera of 200 SLE patients (mean age 34±10.3 years; 26 male and 174 female; median duration of disease 115 months, range 7–378), and of 206 controls, including 19 Sjögren's syndrome, 27 rheumatoid arthritis, 26 psoriatic arthritis, 15 idiopathic inflammatory myopathies (IIM), 13 systemic sclerosis, 49 infectious diseases and 57 healthy subjects, were tested for anti-dsDNA IgG, IgM and IgA isotypes.

**Results:**

Selecting a cutoff corresponding to 95% specificity, the sensitivity of IgG, IgM and IgA anti-dsDNA antibodies in SLE was 55%, 30% and 49%, respectively; 12.5%, 1% and 7.5% of SLE patients had positive IgG, IgM or IgA isotype alone, respectively. SLE patients with glomerulonephritis showed higher levels of IgA anti-dsDNA (p = 0.0002), anti-dsDNA IgG/IgM (p = 0.001) and IgA/IgM (p<0.0001) ratios than patients without renal disease. No significant associations have been found between anti-dsDNA isotypes and other clinical features. IgA anti-dsDNA (p = 0.01) (but not IgG or IgM) and IgG/IgM ratio (p = 0.005) were significantly higher in patients with more active disease (ECLAM score >4).

**Conclusions:**

The detection of IgA anti-dsDNA autoantibodies seems to improve our ability to diagnose SLE and to define lupus nephritis phenotype and active disease. By contrast, IgM anti-dsDNA antibodies might be protective for renal involvement. These data support the hypothesis that anti-dsDNA antibody class clustering may help to refine SLE diagnosis and prognosis.

## Introduction

Anti-double stranded DNA (anti-dsDNA) antibodies are a useful tool for the diagnosis of systemic lupus erythematosus (SLE) [1], [2] and represent one of the criteria of the American College of Rheumatology (ACR) for the classification of SLE. Several studies have shown a correlation between disease activity and anti-dsDNA antibody levels in SLE, particularly in patients with renal involvement [3]–[7], making detection of such antibodies relevant in SLE monitoring [8]. In addition, Belimumab, an anti-B Lymphocyte stimulator monoclonal antibody, was recently approved by the European Medicines Agency (EMA) for SLE patients with active disease as demonstrated by positive anti-dsDNA and C3 or C4 decrease.

However, anti-dsDNA antibodies differ with respect to isotype, avidity, charge, idiotypes and V region sequences [9]. In most SLE patients, IgG-class anti-dsDNA antibodies predominate and they represent the reference antibodies for disease diagnosis. IgG-class anti-dsDNA have also been implicated in the pathogenesis of organ manifestation of SLE, particularly glomerulonephritis, as shown in murine models, where the transfer of murine monoclonal IgG antibodies or anti-dsDNA producing hybridomas into mice induces lupus-like glomerulonephritis [10], [11].

In contrast, anti-dsDNA antibodies of the IgM isotype seem less specific for SLE, and their pathogenic relevance has yet to be elucidated. Some authors demonstrated that IgM anti-dsDNA antibodies does not correlate with disease activity, and no clinical associations have been established [12], [13]. More recently, a negative correlation between IgM anti-dsDNA and glomerulonephritis has been reported [14], [15] and a protective role of IgM anti-dsDNA against immune complex-mediated organ damage has been suggested [16]–[19].

Until now only a few studies evaluated the role of IgA anti-dsDNA in diagnosing and monitoring SLE, and results are conflicting. In fact, some authors reported an association with kidney and joint abnormalities [20], whereas others were not able to demonstrate these associations [21], [22]. Finally, some authors showed a correlation of IgA anti-dsDNA antibodies with vasculitis and acral necrosis, and with some indexes of disease activity such as elevated erythrocyte sedimentation rate and decreased C3 serum levels [21].

The aim of our study was to evaluate the role of the IgG, IgM and IgA isotypes in the diagnosis of SLE, and their association with clinical features and disease activity, in a large cohort of SLE patients, using isotype-specific ELISA assays based on human recombinant dsDNA as antigen source.

## Materials and Methods

### Ethics Statement

The study was approved by the local Ethical Committee of Azienda Ospedaliera di Padova and written informed consent was obtained from each patient.

### Patients

The sera of 200 SLE patients (mean age ± SD 34±10.3 yrs; 26 male and 174 female; median duration of disease 115 months; range 7–378) diagnosed according to ACR criteria [23] were studied. Patients were consecutively enrolled, and clinical data at the time of blood drawing were retrieved from medical records (Table 1). Global SLE activity was measured by the European Consensus Lupus Activity Measure (ECLAM) score [24], and the classification of lupus glomerulonephritis was based on the International Society of Nephrology/Renal Pathology Society (ISN/RPS 2003) classification [25]. Among SLE patients affected with lupus nephritis, 5 had class I/II nephritis (mesangial involvement), and 74 class III/IV (focal or diffuse proliferative lupus nephritis). One patient displaying class V was not included in the analysis while two patients affected with class IV/V were included in the III/IV group for statistical comparison.

**Table 1:** Clinical features and autoantibody profile in 200 SLE patients.

Clinical characteristics		
Sex (M/F)	26/174	
Disease duration median (range) in months	115 (7–378)	
ECLAM score median (range)	3 (0–8)	
**Organ involvement**	**number**	**%**
Glomerulonephritis	82	41
Central nervous system involvement	12	6
Skin rashes	79	39.5
Arthritis	75	37.5
Serositis	17	8.5
Hematological manifestations	112	56
Anemia	13	6.5
Haemolitic anemia	7	3.5
Leukopenia	42	21
Lymphopenia	75	37.5
Thrombocytopenia	24	12
Thromboembolism	35	17.5
**Autoantibody profile**		
ANA positivity	200	100
Anti-dsDNA	134	67
Anti-Sm	37	18.5
Anti-U1RNP	82	41
Anti-SSA	79	39.5
Anti-SSB	25	12.5
IgG anti-cardiolipin	105	52.5
IgM anti-cardiolipin	59	29.5
IgG anti-β2GPI	58	29
IgM anti-β2GPI	38	19
LAC	50	25
**Medications**		
Corticosteroids	162	81
Antimalarials	156	78
Immunosuppressants	92	46
ASA	59	29.5
Warfarin	35	17.5

M: male; F: female; ECLAM: European Consensus Lupus Activity Measurement; ANA: antinuclear antibodies; anti-Sm: anti-Smith; anti-dsDNA: anti-double stranded DNA; anti-β2GPI: anti-Beta 2 glicoprotein I; LAC: lupus anticoagulant; ASA: Aspirin.

In addition, 206 control sera from 19 patients with Sjögren’s syndrome (American-European Consensus Classification Criteria [26]), 27 with rheumatoid arthritis (ACR/EULAR criteria [27]), 26 with psoriatic arthritis (diagnosed according to clinical, radiological and synovial fluid findings in patients affected with psoriasis or with a first-grade relative affected), 15 with idiopathic inflammatory myopathies (IIM; Bohan and Peter’s criteria [28], [29]), 13 with systemic sclerosis (ACR criteria [30]), 49 with infectious diseases (13 hepatitis B virus; 10 cytomegalovirus; 10 Epstein Barr virus; 16 hepatitis C virus), and 57 from healthy subjects were tested to determine the diagnostic accuracy of the anti-dsDNA assays. Sera of patients affected with rheumatologic disorders were collected in the Rheumatology Unit of Padua University and sera of patients with infectious diseases and healthy subjects were collected in the Allergy and Clinical Immunology Unit of the Pordenone Hospital. Sera of patients with infectious diseases and healthy controls were collected from February 2009 to September 2010, sera of SLE patients and patients affected with other autoimmune rheumatic diseases were collected from 2000 to 2009. All sera were frozen at −80°C until they were processed.

### Anti-dsDNA Measurement

Anti-dsDNA IgG, IgM and IgA isotypes were measured according to manufacturer’s instructions, by commercially available ELISA assays (Aeskulisa dsDNA-G, Aeskulisa dsDNA-M, Aeskulisa dsDNA-A; Aesku Diagnostic, Wendelsheim, Germany), based on a human recombinant dsDNA source as antigen bound to microwells. Briefly, 10 µL of diluted sera (1∶100) were incubated for 30 minutes on the ELISA plates, and after three washing steps, the horseradish peroxidase labeled antihuman-IgG (rabbit), -IgM (goat) or –IgA (goat) was added as a conjugate and incubated at room temperature for 15 minutes. After three additional washing steps, 100 µL of tetramethylbenzidine (TMB) were added and the samples were further incubated for 15 minutes before stopping the reaction with 100 µL 1M HCL. Optical density (OD) was measured at 450 nm. All assay procedures were performed automatically on the Triturus® (Grifols, Barcelona, Spain) microplate analyzer. The values were expressed in International Units (IU)/mL for IgG, calibrated against the Wo/80 reference standard, and in U/mL for IgM and IgA anti-dsDNA, as no international standard for these antibody isotypes is available.

Cut-offs for IgG, IgM and IgA anti-dsDNA antibodies, selected at 95% specificity as determined by receiver operating characteristic (ROC) curve analysis, were 73.5 IU/mL, 69.5 U/mL and 28.0 U/mL, respectively. The ROC curves were built considering all the non-SLE sera as variable indicating diagnosis different from SLE.

### Statistical Analysis

Associations between non-parametric unpaired data were calculated by means of the Mann- Whitney U test and data were adjusted using the Bonferroni’s test for multiple comparisons. Prevalence of different variables were compared using chi-square (χ^2^) test. All tests were used with two-sided options and significance level was set at a p value <0.05. SPSS for Windows, version 11.5 (SPSS Inc., Chicago, IL) and MedCalc for Windows, version 7.4.19 (MedCalc software, Mariakerke, Belgium) were used for the statistical analysis.

## Results

**Figure 1:**
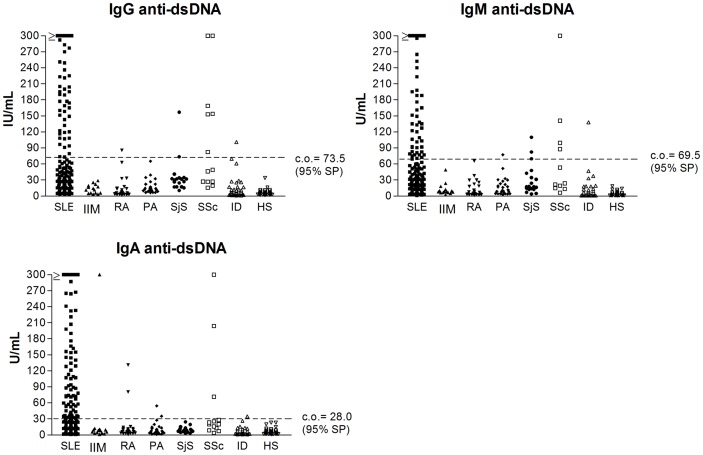
Distribution of anti-dsDNA IgG, IgM and IgA isotype values in SLE and controls. Footnotes: IU: international units; IgG: immunoglobulins class G; IgM: immunoglobulins class M; IgA: immunoglobulins A; anti-dsDNA: antibodies against double stranded DNA; SLE: systemic lupus erythematosus; IIM: idiopathic inflammatory myopathies; RA: rheumatoid arthritis; PA: psoriatic arthritis; SjS: Sjögren’s syndrome; SSc: systemic sclerosis; ID: infectious diseases; HS: healthy subjects; c.o. = cut off; SP: specificity.

Setting the same specificity value for all isotypes at 95% by ROC curves, sensitivity of IgG, IgM and IgA anti-dsDNA antibodies in SLE was 55%, 30% and 49%, respectively. As shown in Table 2, 12.5% of SLE patients resulted positive for the IgG isotype alone, whereas 1% and 7.5% were positive for the IgM and IgA isotype alone, respectively. Considering all three antibody classes, the sensitivity increases to 67%, but the specificity decreases to 90.7%. The distribution of class-specific anti-dsDNA levels are shown in Figure 1.

**Table 2:** Prevalence of different anti-dsDNA antibody isotypes in 200 SLE patients.

Anti-dsDNA isotype	positive	%
Total IgG	110/200	55%
Total IgM	60/200	30%
Total IgA	98/200	49%
IgG alone	25/200	12.5%
IgM alone	2/200	1%
IgA alone	15/200	7.5%
IgG+IgM+IgA	42/200	21%
IgG +IgM	9/200	4.5%
IgG+IgA	34/200	17%
IgA+IgM	7/200	3.5%
Overall anti-dsDNA positivity	134/200	67%

Anti-dsDNA: antibodies against double stranded DNA; IgG: immunoglobulins class G; IgM:

immunoglobulins class M; IgA: immunoglobulins class A.

**Figure 2:**
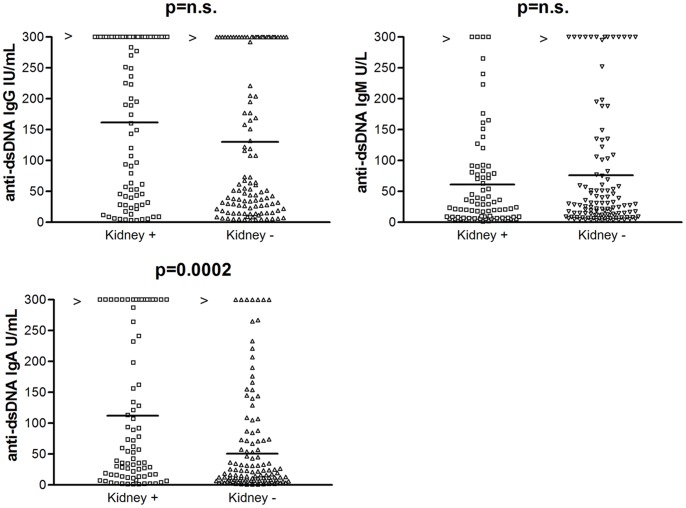
Comparison of anti-dsDNA IgG, IgM and IgA autoantibody levels in SLE patients with and without kidney involvement. Median serum values are illustrated (horizontal lines). Median serum values (horizontal lines) of IgG, IgM and IgA anti-dsDNA resulted 171.0±125.1 IU/mL (range: 2.8–2046); 59.5±78.8 U/mL (range: 1.0–503), 122.5±122.8 U/mL (range: 1.0–1012) in patients with kidney involvement, and 131±123.7 IU/mL (range: 3.0−1018), 76.1±100.5 U/mL (range: 2.6–718), 49.8±84.7 U/mL (range: 1.0–382) in patients without kidney involvement, respectively. Footnotes: IU: international units; IgG: immunoglobulins class G; IgM: immunoglobulins class M; IgA: immunoglobulins A; anti-dsDNA: antibodies against double stranded DNA.

**Figure 3:**
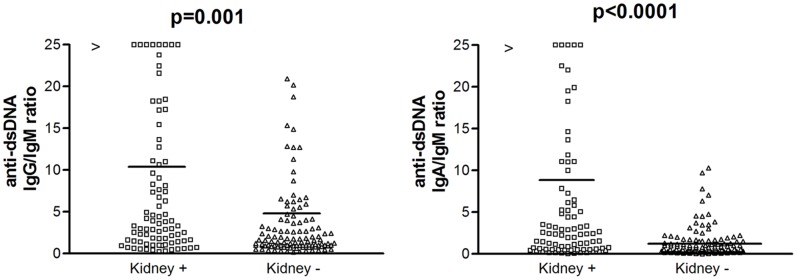
Anti-dsDNA IgG/IgM and IgA/IgM ratios in SLE patients with (10.8±23.8, range 0.25−160 and 9.36±7.3, range 0.4–28, respectively) and without (4.6±7.8, range 0.18–22.6 and 1.51±1.8, range 0.01–10.3, respectively) kidney involvement. Median serum values are shown (horizontal lines). Footnotes: IgG: immunoglobulins class G; IgM: immunoglobulins class M; anti-dsDNA: antibodies against double stranded DNA.

No significant difference was found in IgG and IgM anti-dsDNA levels in patients with or without kidney involvement. Conversely, patients with kidney involvement showed higher levels of IgA anti-dsDNA (p = 0.0002) (Figure 2), and a significant higher frequency of lupus nephritis has been shown in IgA anti-dsDNA positive (56/98, 57%) in respect to IgA anti-dsDNA negative (27/102, 26%) (χ^2^ = 19.3; p = 0.0001). However, no significant difference in the frequency of glomerulonephritis was shown in patients positive for IgA anti-dsDNA alone (5/15, 33.3%) vs. IgA anti-dsDNA negative (27/102, 26%) (χ^2^ = 0.31; p = 0.577), in patients positive for IgA anti-dsDNA alone (5/15, 33.3%) vs. anti-dsDNA negative (15/66, 23%) (χ^2^ = 0.74; p = 0.389), as well as in patients positive for IgG anti-dsDNA alone (5/25, 20%) vs. IgG anti-dsDNA negative (27/90, 30%) (χ^2^ = 0.97; p = 0.323) and in patients positive for IgG anti-dsDNA alone vs. anti-dsDNA negative (15/66, 23%) (χ^2^ = 0.08; p = 0.779). On the other hand, a higher frequency of kidney involvement has been shown in patients positive for both IgG and IgA anti-dsDNA (45/76, 59%) vs. IgG and IgA anti-dsDNA negative (37/124, 30%) (χ^2^ = 16.8; p = 0.0000). It is interesting to remark as no significant difference in kidney involvement has been shown in patients positive for all IgG, IgA and IgM anti-dsDNA isotypes (19/23, 83%) vs. negative subjects (18/44, 41%) (χ^2^ = 2.87; p = 0.09). Moreover, in SLE patients with renal disease, anti-dsDNA IgG/IgM and IgA/IgM ratios resulted significantly higher than in patients without renal disease (p = 0.001 and <0.0001, respectively) (Figure 3). No significant difference was found in the three anti-dsDNA isotypes between SLE patients with lupus gomerulonephritis of classes I/II and III/IV. We also evaluated whether the IgG/IgM and the IgA/IgM anti-dsDNA ratios could be used to assess renal involvement in SLE patients. Based on ROC analysis, the optimal IgG/IgM anti-dsDNA ratio value was 2.09 (sensitivity = 64.6%, 95%CI 53.3–74.9%; specificity = 60.2%, 95%CI 50.7–68.1%; positive likelihood ratio [LR+] = 1.62; negative likelihood ratio [LR-] = 0.59); the IgA/IgM anti-dsDNA ratio value was set at 1.74 (sensitivity = 54.9%, 95%CI 43.5–65.9%; specificity = 83.9%, 95%CI 76.0–90%; LR+ = 3.41; LR- = 0.54). However, the area under the ROC curve was low for both the IgG/IgM ratio (0.633) and the IgA/IgM ratio (0.693).

**Figure 4:**
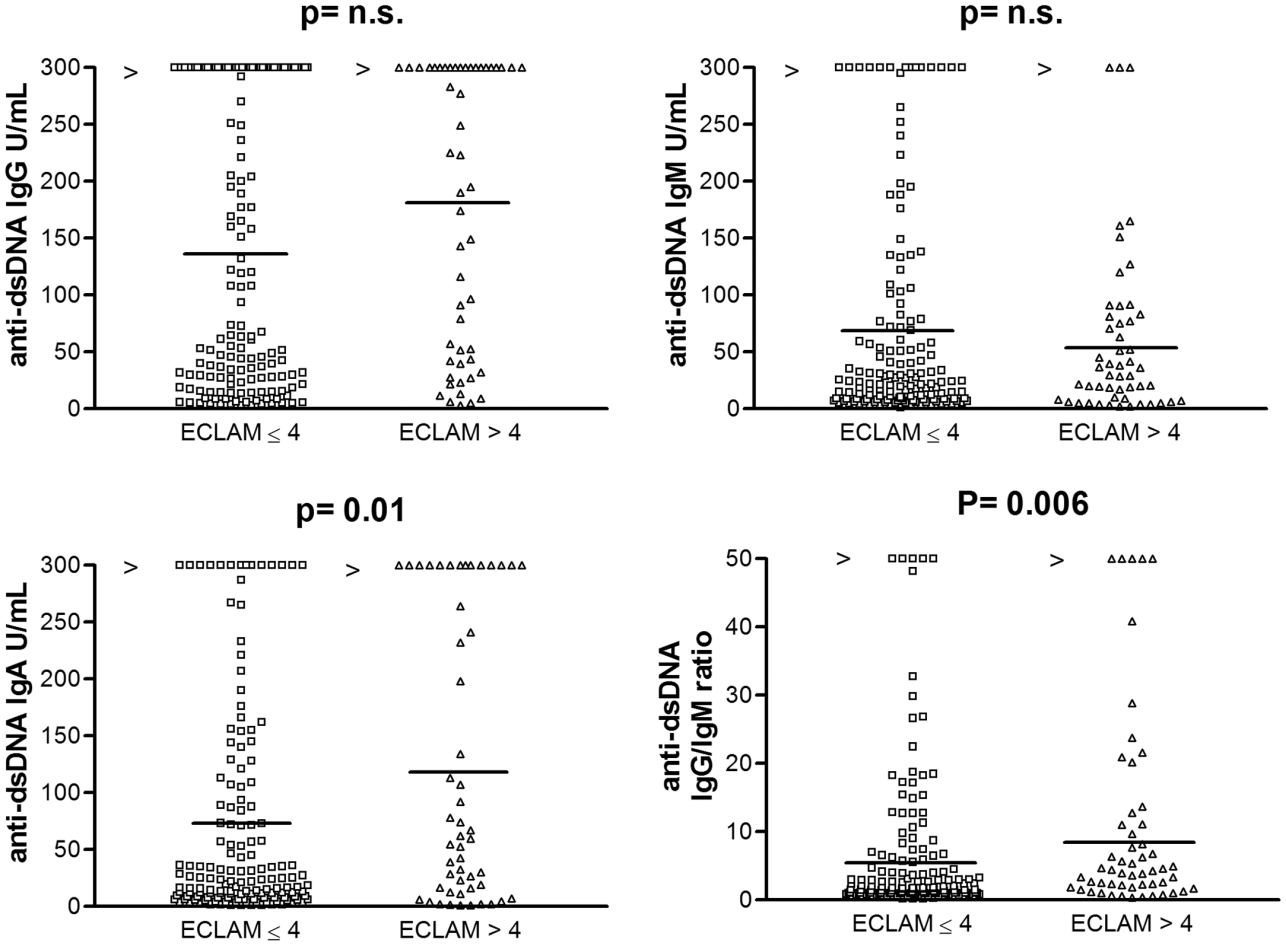
IgG, IgM and IgA anti-dsDNA isotypes levels and IgG/IgM anti-dsDNA ratio in patients with ECLAM score ≤4 (138.8±124.6 IU/mL, range: 2.8–901; 72.7±97.6 U/mL, range: 1.0–1012; 72.8±96.6 U/mL, range: 1.0−543; and 6.25±10.6, range 0.18–83, respectively) and with ECLAM score >4 (175±122.4 IU/mL, range: 2.9–2046; 58.9 U/mL ±74.6, range 1.0–618; 124.6±124.7 U/mL, range 1.0–1018, and 10.7±14.9, range 0.32–103, respectively). Association between IgG, IgM and IgA anti-dsDNA isotypes and IgG/IgM anti-dsDNA ratio and the ECLAM score. Median serum values are shown (horizontal lines). Footnotes: IU: international units; IgG: immunoglobulins class G; IgM: immunoglobulins class M; IgA: immunoglobulins A; anti-dsDNA: antibodies against double stranded DNA; ECLAM: European Consensus Lupus Activity Measurement.

No significant associations have been found between anti-dsDNA isotypes and other clinical features of organ damage (skin, serosa, musculoskeletal, central nervous system, hematological manifestations and thromboembolism) (p>0.016). IgA anti-dsDNA (p = 0.01) (but not IgG or IgM) and IgG/IgM anti-dsDNA ratio (p = 0.006) were significantly higher in patients with more active disease (ECLAM score >4) (Figure 4). A significant higher frequency of patients with ECLAM score >4 has been shown in IgA anti-dsDNA positive (35/98, 36%) in respect to IgA anti-dsDNA negative patients (16/102, 16%) (χ^2^ = 10.55; p = 0.0012), as well as in patients positive for IgA anti-dsDNA alone (8/15, 54%) versus IgA anti-dsDNA negative (28/102, 25%) (χ^2^ = 4.11; p = 0.042), in patients positive for IgA anti-dsDNA alone (8/15, 54%) vs. anti-dsDNA negative (12/66, 18%) (χ2 = 8.12; p = 0.004), and in patients both IgG and IgA anti-dsDNA positive (45/76, 59%) vs. IgG and IgA dsDNA negative (25/124, 20%) (χ^2^ = 31.58; p = 0.0000). Only a slight association has been shown by testing patients positive for IgG anti-dsDNA alone (10/25, 40%) vs. anti-dsDNA negative (13/66, 20%) (χ^2^ = 3.96; p = 0.0467), whereas no association has been shown comparing positive for IgG anti-dsDNA alone (10/25, 40%) vs. IgG anti-dsDNA negative (19/90, 21%) (χ^2^ = 3.7; p = 0.0544). Finally, a significant higher frequency of patients with ECLAM score >4 has been shown in patients positive for all anti-dsDNA isotypes (12/30, 40%) compared with negative patients (7/59, 12%) (χ^2^ = 5.51; p = 0.016).

## Discussion

In this study we evaluated the diagnostic accuracy of IgG, IgM and IgA anti-dsDNA antibody isotypes and their clinical relevance in predicting organ damage, particularly renal involvement, in SLE patients. Fixing for each antibody class a specificity of 95%, we confirmed that in SLE the prevalent anti-dsDNA antibody isotype is represented by IgG (55%). However, 49% of SLE patients were positive for IgA anti-dsDNA, and in 7.5% of the cases it was the only anti-dsDNA isotype present. These results seem in contrast with those previously reported by Atta et al. in a subset of Brazilian SLE patients [22], where IgA anti-dsDNA antibodies were detected only in 19.4% of the subjects and were always associated with IgG anti-dsDNA antibodies. This disagreement may be attributed to the different methods used for anti-dsDNA detection [31] and to the different genetic background of the SLE populations studied [2]. Indeed, Förger et al [15], using the same method used in our study in a large cohort of German SLE patients, showed a prevalence of IgA anti-dsDNA of 40%. In the same study, however, the prevalence of IgG and IgM anti-dsDNA was higher (80% and 57%, respectively) than in our study. This may be attributed to the different cut-offs used. In fact, Förger et al. used the cut-off suggested by manufacturer (20 U/mL for all anti-dsDNA isotypes), whereas in our study, cut-off corresponding to 95% specificity (73.5 IU/mL for IgG, 69.5 U/mL for IgM and 28 U/ml for IgA) were selected. Therefore, in our study the best combination of sensitivity (62.5%) and specificity (92.8%) for SLE diagnosis was achieved by the combination of IgG and IgA anti-dsDNA. In this regard, it has to be mentioned that high levels of anti-dsDNA antibodies found in some SSc patients may be related to intrinsic ELISA test features, depending on the method and the cut-off used [32]–[35], although antihistone, antinucleosome and even anti-dsDNA antibodies have actually been detected in a proportion of SSc patients by ELISA assays. Cross-reactivity also has to be considered, since the CENP-A centromere protein has been shown to be a centromere-specific histone H3 variant [36], therefore SSc sera might exhibit cross-reactive recognition of chromatin structures, including dsDNA, mediated by primary antihistone immunological reactivities.

It is generally believed that IgG anti-dsDNA autoantibodies play an important role in the pathogenesis of SLE in particular in the induction of nephritis, and IgG anti-dsDNA antibodies and immune-complexes are detectable in the glomeruli of patients with lupus nephritis [18], [19], [37], [38].

However, some authors were not able to demonstrate a significant association of IgG anti-dsDNA with kidney involvement or disease activity [39]. It has been suggested that it may depend on the method used for anti-dsDNA detection [35], as methods detecting mainly high avidity IgG antibodies seem to supply a better correlation between anti-dsDNA levels and disease activity and kidney involvement [40]. However, in a previous work we were not able to demonstrate a correlation between disease activity and anti-dsDNA avidity [41]. On the other hand, IgM anti-dsDNA antibodies seem to be a protective factor for glomerulonephritis development, likewise IgG anti-pentraxin 3 antibodies [18], [42], both in animal models [43], [44] and in SLE patients [17], [21]. This finding may be related to the greater ability of IgM class antibodies to bind more circulating antigen, thus decreasing via competitive inhibition the formation of IgG-class immune complexes. Another potential explanation is that IgM anti-dsDNA antibodies may down-regulate autoreactive B cells and decrease the production of pathogenetic IgG anti-dsDNA antibodies. In addition, it has been suggested that IgM anti-dsDNA-dsDNA complexes are cleared more effectively by phagocytes and therefore minimally deposited in the glomerular basal membrane [16], [18]. In our study, although no significant difference has been found in the levels of IgM anti-dsDNA in patients with and without kidney involvement, a high IgG/IgM ratio was significantly associated with glomerulonephritis. These findings confirm the results obtained by Förger et al. [15], though the IgG/IgM ratio value combining both the highest sensitivity and specificity for renal involvement was higher than in Förger study (2.09 vs 0.8). The IgG/IgM anti-dsDNA ratio was also significantly higher in patients with active disease (ECLAM >4), confirming that IgM-class antibodies might exert a protective role.

Interestingly, IgA anti-dsDNA autoantibodies levels were significantly associated with both glomerulonephritis and active disease. These results are in agreement with those obtained by Miltenburg et al. [20], and only partially with those obtained by Witte et al. [21], who found an association with disease activity, but not with glomerulonephritis. However, since the strongest association with lupus nephritis and active SLE was observed when both IgA and IgG anti-dsDNA isotypes were present, detection of both isotypes is likely to be superior and more useful than detection of only one isotype in diagnosis of lupus nephritis and active SLE. Although IgA antibodies are unable to activate the classical complement pathway, recent insights suggest they may be implicated in the activation of the alternative complement pathway in lupus glomeruli [45], notwithstanding the fact that no antibody binding is usually required to such pathway. However, IgA anti-dsDNA may somehow elicit non-classical complement activation and worsen renal damage in SLE [46]. The results of the studies concerning the association between IgA anti-dsDNA and other clinical features, including arthritis, are inconclusive [20], [21]. Further studies in large cohorts of SLE patients are needed to evaluate the association between IgA anti-dsDNA and other clinical manifestations such as skin, central nervous system or serosa involvement, vasculitis, thromboembolism and hematological manifestations.

We notice a limitation in our study, since antibodies were only measured in sera while they were not searched on kidney biopsies; however, this is consistent with the need to pinpoint serological biomarkers for disease activity and phenotypes. In conclusion, our study confirmed the role of IgG anti-dsDNA antibodies in the diagnosis of SLE and suggests that the detection of IgA anti-dsDNA antibodies can improve our ability to diagnose SLE (7.5% of SLE patients were positive only for this autoantibody class), particularly lupus nephritis. By contrast, IgM anti-dsDNA antibodies might be protective for renal involvement. The afore-mentioned data support the hypothesis that anti-dsDNA antibody class clustering may help to refine SLE diagnosis and prognosis.
